# Can decision support combat incompleteness and bias in routine primary care data?

**DOI:** 10.1093/jamia/ocab025

**Published:** 2021-03-11

**Authors:** Olga Kostopoulou, Christopher Tracey, Brendan C Delaney

**Affiliations:** Department of Surgery and Cancer, Imperial College London, St Mary's Campus, Norfolk Place, London, UK

**Keywords:** primary health care, documentation, electronic health records, bias, decision support systems, clinical

## Abstract

**Objective:**

Routine primary care data may be used for the derivation of clinical prediction rules and risk scores. We sought to measure the impact of a decision support system (DSS) on data completeness and freedom from bias.

**Materials and Methods:**

We used the clinical documentation of 34 UK general practitioners who took part in a previous study evaluating the DSS. They consulted with 12 standardized patients. In addition to suggesting diagnoses, the DSS facilitates data coding. We compared the documentation from consultations with the electronic health record (EHR) (baseline consultations) vs consultations with the EHR-integrated DSS (supported consultations). We measured the proportion of EHR data items related to the physician’s final diagnosis. We expected that in baseline consultations, physicians would document only or predominantly observations related to their diagnosis, while in supported consultations, they would also document other observations as a result of exploring more diagnoses and/or ease of coding.

**Results:**

Supported documentation contained significantly more codes (incidence rate ratio [IRR] = 5.76 [4.31, 7.70] *P *<* *.001) and less free text (IRR = 0.32 [0.27, 0.40] *P *<* *.001) than baseline documentation. As expected, the proportion of diagnosis-related data was significantly lower (b = −0.08 [−0.11, −0.05] *P *<* *.001) in the supported consultations, and this was the case for both codes and free text.

**Conclusions:**

We provide evidence that data entry in the EHR is incomplete and reflects physicians’ cognitive biases. This has serious implications for epidemiological research that uses routine data. A DSS that facilitates and motivates data entry during the consultation can improve routine documentation.

## INTRODUCTION

Documenting the clinical consultation in the electronic health record (EHR) serves several purposes in addition to direct clinical care. The commonest are billing (especially in the US) and the monitoring of performance targets, such as those used in the UK Quality and Outcomes Framework (also connected to remuneration).^1^ Other purposes include service quality improvement and epidemiological research. This article is concerned with the last purpose and primarily the use of EHR-derived data in developing evidence to support diagnosis.

Clinical codes are an agreed set of terms that capture individual pieces of clinical information. The intention is to reduce ambiguity in clinical documentation, enable analysis of clinical data, and support maintenance of problem lists, disease registers, automated functions, and alerts and reminders in an EHR system. The concept of coding was introduced to the UK National Health Service in 1985.^2^ In the UK, data from the Clinical Practice Research Database (CPRD), QResearch, ResearchOne, and the Royal College of General Practitioners’ Research and Surveillance Centre (RSC) contain coded data extracted from primary care EHRs. They have all been used widely in research and have become more useful since being linked to data from other parts of the health system. In the US, the Patient Centered Outcomes Research Network (PCORNet), a network of regional data repositories, has opened up EHR data for researchers.^3^ EHR data have been used to derive cardiovascular, cancer, and diabetes prediction models,^4^^,^^5^ to recruit patients to clinical trials, and to explore disease prevalence and demand for healthcare. EHR data have also been used to derive diagnostic prediction rules where the relationships between symptom codes and diagnostic labels can be explored.

Evidence-based diagnosis depends on knowing the predictive value of a given symptom or sign for the diagnosis in question. Where several clinical facts need to be combined, adjustment for nonindependence is made using a clinical prediction rule. Similar to treatment and prognosis, it is efficient to use routinely documented clinical data. Most errors in the EHR are errors of omission, are more likely in the documentation of the presenting complaint, and have been linked to the way the EHR is structured for billing purposes.^6^ However, bias in the way that general practitioners (GPs) document symptoms and signs during the consultation has not been investigated. It is generally assumed that GPs record what they observe and what the patient recounts. Below, we explain why this may not be the case. We should also note that the term “bias” can have different meanings in different contexts. We use the term both to refer to “cognitive biases,” patterns of reasoning that depart from rationality,^7^ and “statistical bias,” errors in inference induced by nonrepresentative data. We will distinguish these clearly in the subsequent text. Cognitive bias can lead to statistical bias by providing a mechanism whereby data are not recorded in a representative fashion.

Firstly, entering data in code requires additional effort and time by the clinician. A GP needs about 30 seconds to enter an item of information in code in existing EHR systems.^8^ Given this additional effort, GPs may code only what they consider important. Indeed, GPs have been found to record certain symptoms in code rather than in free text more frequently for cancer than noncancer patients.^9^ This suggests a preference towards the coding of symptoms that the physician considers to be related to the diagnosis, while free-texting symptoms he/she considers unrelated—a source of potential statistical bias in epidemiological research that uses coded data. Can we, however, rest assured that the sum of free text and codes in the clinical record provides a complete account of how the patient presented? Again, this assumption can be called into question.

Secondly, most GPs do not record in real time but at the end of the consultation, often when the patient has left the room.^10^ Anecdotal evidence from the US suggests that recording may take place at the end of the day.^11^ Delayed recording can degrade documentation by introducing memory gaps and distortions: clinicians may not remember everything that they observed during the consultation but only what they consider important and what supported their diagnosis or decision. In addition to memory gaps, there is a tendency for decision consolidation: after we make a decision, we tend to restructure facts and reappraise information so that it supports the decision made.^12^^,^^13^ This reduces the uncertainty that we experienced at the time preceding the decision but also fortifies the decision against future threats. For example, GPs may feel the need to justify their diagnosis, safeguarding it against potential challenges; this need can be served by preferentially entering information that fits with the diagnosis.

Finally, even if GPs attempt to document in real time, the information may be limited by their working hypothesis, and aspects of the patient’s illness experience may remain unexplored. To prevent physicians from focusing on 1 hypothesis too quickly, ignoring other possibilities, we have previously designed a prototype decision support system (DSS) for diagnosis in primary care,^14^^,^^15^ which we evaluated in simulated consultations with GPs and standardized patients (actors).^10^^,^^16^ The main feature of the DSS is to suggest to GPs possible diagnoses to consider at the start of the consultation, based on patient demographics, risk factors, and the presenting problem.^10^ In our evaluation study, we found that the DSS improved diagnostic accuracy without significantly increasing the number of investigations. Furthermore, we measured a significant increase in coding, from 1.6 to 12.4 items per consultation on average.^10^

The DSS was designed to facilitate coding in real time. As users start typing in a symptom, a drop-down menu appears, which presents them with various options to choose from, and the system allocates the right code in the background. Users can also select from a list of symptoms and signs related to each of the suggested diagnoses and indicate whether they are present or absent. Thus, users never need to search for codes. Furthermore, the DSS motivates users to code during the consultation, because they can interact with it: they see the order of the suggested diagnoses change on their screen as the coded symptoms and signs accumulate.^16^

The impressive increase in coding with the DSS inspired the present investigation. This involved analysis of a dataset from the DSS evaluation study that has not been analyzed before, namely, the documentation of the participating GPs, with 2 aims: 1) to determine whether using the DSS leads not only to more coding but to recording more information in general; and 2) to ascertain whether GPs preferentially record information related to their final diagnosis, and if this tendency is reduced when using the DSS.

### The DSS evaluation study: study design and diagnostic scenarios

In the study, 34 GPs consulted with 12 standardized patients (SPs). In a within-participant design, GPs took part in 2 sessions. In the first session, they consulted with 6 SPs using their usual EHR (baseline consultations). In the second session, on a different day, they consulted with 6 other SPs using the EHR-integrated DSS (supported consultations).

We prepared 12 detailed clinical scenarios for the SPs. Most scenarios had been used in previous studies by Kostopoulou, Delaney, and colleagues^15^^,^^17^^,^^18^ and covered a wide range of diagnostic difficulty from low to high. All scenarios consisted of a patient description (demographics, risk factors, past medical history, medications, presenting problem) and a long list of signs and symptoms, which served to answer any questions GPs might ask the patient. The number of information items ranged between 34 and 56 depending on the scenario. The total information available in each scenario script allowed for only a single correct diagnosis.

Scenarios were counterbalanced across GPs, so that each scenario was seen with and without the DSS an equal number of times. For the counterbalancing, we split the 12 scenarios in 2 blocks of 6, which we distributed equally between the 2 consultation modes. Consequently, not all GPs saw the same patient in the same consultation mode. For example, GP1 saw patient 3 at a baseline consultation, while GP2 saw patient 3 with the DSS.

The clinical notes for each consultation, including coded and free-text elements, were automatically recorded in the EHR, and were thus available for analysis. The charts from a total of 408 (34 x 12) consultations were available. Here, we compare the documentation (number and type of data items) between baseline and supported consultations. The baseline consultations, which were conducted using the GP’s usual EHR system, served as a control, reflecting the GPs’ usual approach to routine documentation.

### Hypotheses

We expect that clinical documentation does not reflect completely and accurately how the patient presented and what the physician observed, unless the physician is both motivated and enabled to record during the consultation. Given the increase in coding with the DSS, we expect that documentation in the supported consultations is more extensive in general. Thus, we hypothesize that more data items will be documented in the EHR in the supported consultations, both in code and free text (*H*_1_). We also expect that documentation in the baseline consultations will be more consistent with the diagnosis that the physician gave, given our drive for decision consolidation and cognitive coherence.^19^^,^^20^ Thus, we hypothesize that a higher proportion of data items related to the final diagnosis is documented in the baseline consultations compared to the supported consultations (*H*_2_).

## MATERIALS AND METHODS

In order to test these 2 hypotheses, we need to know what clinical facts were available in the scenarios depicted by the actors, what information each GP documented in the EHR in either code or free text, and what diagnoses each GP made.

### Script data: the reference standard

**Figure 1.**
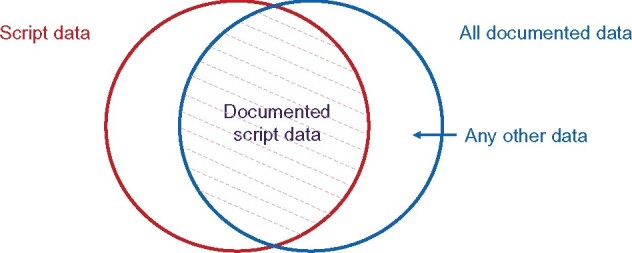
A pictorial representation of the types of data analyzed.

Each scenario script was broken down to individual information items, such as risk factors, symptoms, and signs. These were the predefined clinical data items, the pool of information that GPs could have elicited from the actors. They are represented by the red circle in Figure 1.

### All documented data

Each GP’s EHR documentation at each consultation was also broken down to individual clinical concepts, which were then annotated by the second author and GP researcher using the reference standard. These annotations were checked by the third author, an academic GP, and no disagreements were found. The EHR documentation is represented by the blue circle in Figure 1. A documented datum could be either part of the reference standard (‘documented script data’—the shaded intersection in Figure 1) or not (‘any other data’). It could be either coded or free text.

**Type of documented script data:** We characterized the documented script data items as either diagnosis-related (ie, related to a GP’s final diagnosis) or not. In order to do this, we followed a modified Delphi Technique. For each scenario, we paired the script data available for elicitation with the list of final diagnoses that the study GPs had given. We gave these paired lists to 5 independent GPs who did not participate in the original study. We asked them to indicate in each scenario which script data were related to each given diagnosis, that is, *which data a GP might reasonably request in relation to a diagnosis*. Script data could be assigned to more than 1 diagnosis. If ≥3 of the 5 GPs agreed that a script datum was related to a diagnosis, this was taken as such.

### Statistical analyses

To regress the number of documented data items on the predictor variable (consultation mode, ie, baseline vs supported), we employed a mixed effects Poisson regression with random intercept by GP and chose the robust standard errors option.^21^ We modeled the coded and free-text data separately. Due to overdispersion, we used mixed effects negative binomial regressions to model the coded and free-text data. According to *H*_1_, we expected more data items to be documented in the supported consultations.

We calculated the proportion of diagnosis-related data items out of all documented data. Proportions were created from count data and could take any value between 0 (there are no diagnosis-related data items amongst the documented data) and 1 (all documented data items are diagnosis-related). We treated the variable as continuous for the analyses. To test the effect of consultation mode (baseline vs supported) on the proportion of diagnosis-related data items, we employed a mixed effects linear regression with random intercept by GP. We ran separate regressions for the coded and free-text data. We performed checks on the normality of the regression residuals using the Shapiro-Wilk test for normality. Where residuals were not normally distributed, we supplemented the regression analyses with the Wilcoxon matched-pairs signed-ranks test and the sign test. According to *H*_2_, we expected supported consultations to contain a lower proportion of diagnosis-related data items than baseline consultations. Stata 13.1 was used for all the analyses.

## RESULTS

Table 1 provides descriptive statistics (means and standard deviations) for the number of documented data, the number of diagnosis-related data, and the proportion of diagnosis-related data by consultation mode (baseline and supported) and data type (code and free text).

**Table 1.** Means (SD) of the variables analysed, by consultation mode and data type

	Baseline Consultations Mean (SD)	Supported Consultations Mean (SD)
1) All documented data	12.15 (4.00)	15.73 (5.22)
1a) All documented codes	2.15 (2.53)	12.39 (5.33)
1b) All documented free text	10.00 (4.24)	3.34 (3.03)
2) Diagnosis-related data	8.40 (2.86)	9.60 (3.36)
2a) Codes	1.49 (1.70)	7.72 (3.42)
2b) Free text	6.92 (3.08)	1.89 (2.14)
3) Bias all data: diagnosis-related script data/all documented data	0.71 (0.17)	0.63 (0.17)
3a) Bias codes: diagnosis-related script codes/all codes	0.78 (0.33)	0.65 (0.19)
3b) Bias free text: diagnosis-related script free text/all free text	0.72 (0.21)	0.54 (0.37)

As hypothesized (*H*_1_), participants documented significantly more data items in the supported than the baseline consultations: IRR = 1.29 [1.18, 1.42] *P *<* *.001. Thus, compared to the baseline consultations, the incidence rate of the documented data in the supported consultations is expected to increase by 1.29, in other words, by almost 30%. For the coded data, the incidence rate is expected to increase by 5.76 [4.31, 7.70] (*P *<* *.001). In contrast, significantly fewer free-text data items were documented in the supported consultations: IRR = 0.32 [0.27, 0.40] *P *<* *.001. Thus, *H*_1_ was only partly supported.

**Figure 2.**
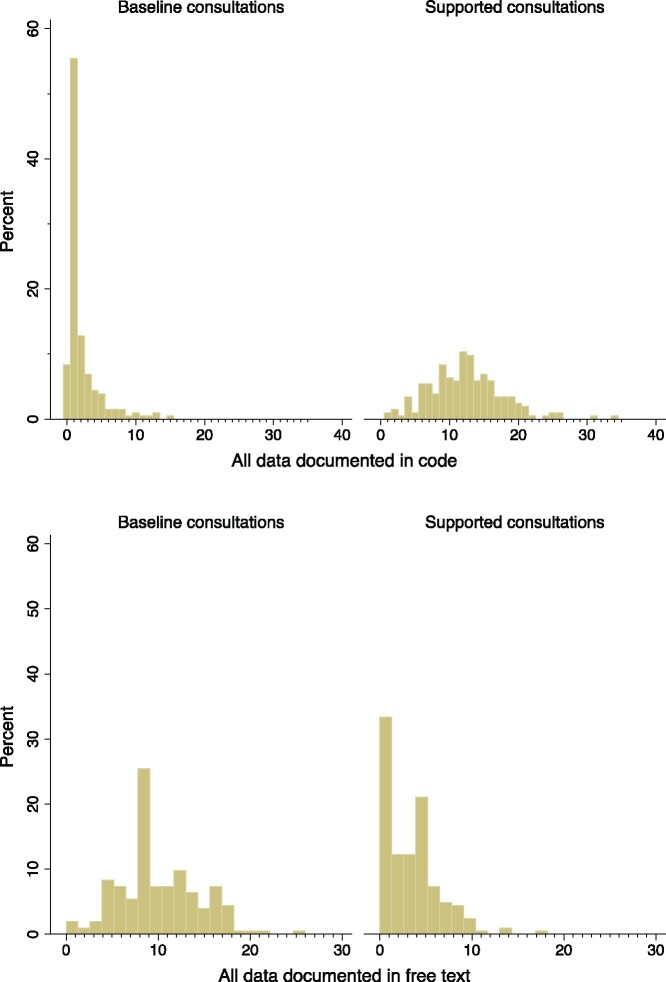
Histogram of the percentage of data documented in code (top panel) and free text (bottom panel) by consultation mode.

Figure 2 shows that in the majority of the baseline consultations, either no codes at all or only 1 code was entered (top panel), while in over 30% of the supported consultations, 1 or fewer free-text entries were made (bottom panel). Thus, the increase in the overall amount of documentation in the supported consultations was due to more data being coded into the EHR.

**Figure 3.**
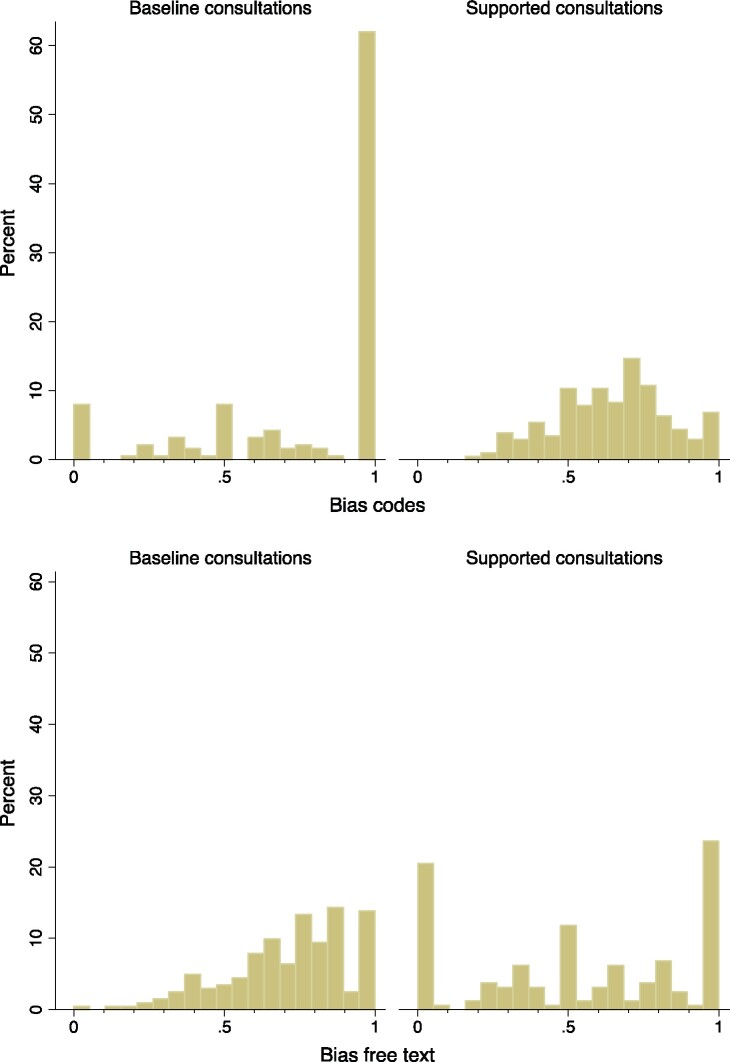
Bias in the documentation by consultation mode. Proportion of diagnosis-related codes out of all codes documented (top panel); proportion of diagnosis-related free-text data items out of all free-text data items documented (bottom panel).

As hypothesized (*H*_2_), the proportion of diagnosis-related data items documented in the EHR out of all data items documented was significantly lower in the supported consultations (b = −0.08 [−0.11–−0.05] *P *<* *.001). This was the case for both codes (b = −0.13 [−0.19–−0.08] *P *<* *.001) and free-text (b = −0.18 [−0.24–−0.12] *P *<* *.001). The top panel of Figure 3 shows that in over 60% of the baseline consultations, all data items coded into the EHR were related to the final diagnosis.

A check on the normality of the regression residuals identified that the residuals from the linear regressions for both codes and free text were not normally distributed. Although this does not affect the regression coefficients, the *P* values may not be valid. Thus, we supplemented the analyses by conducting Wilcoxon matched-pairs signed-ranks tests, which test the null hypothesis that the distributions are the same. We also conducted a sign test, which tests the equality of matched pairs of observations. The null hypothesis is that the median of the differences is zero; no further assumptions are made about the distributions. To conduct these tests, we created new variables that contained only average values per GP. Specifically, for each of the 34 GPs and per consultation mode, we averaged 1) the proportion of coded data items that were related to the final diagnosis and 2) the proportion of free-text data items that were related to the final diagnosis (3a and 3b in Table 1). We thus created 4 new variables of 34 values each (two variables per consultation mode), which we then tested using the Wilcoxon matched-pairs signed-ranks test and the sign test for matched pairs. The Wilcoxon test rejected the null hypothesis that the distributions of 1) the proportion of coded data items (z = 3.08, *P *=* *.002) and 2) the proportion of free-text data items (z = 4.19, *P *<* *.001) were the same when comparing baseline with supported consultations. One-sided sign tests showed that the median of the difference (Baseline—Supported) for the proportion of coded data items was significantly larger than 0 (*P *=* *.03) and so was the difference for the proportion of free-text data items (*P *=* *.0002).

## DISCUSSION

Our study shows that routine clinical documentation is incomplete and that the documented information, whether in free text or code, relates mainly to the diagnosis given at the consultation—suggesting that cognitive bias has driven selective recording of clinical facts. This selective recording will result in statistical bias of the data obtained from EHR systems for use in epidemiological research, undermining the validity of diagnostic tools and risk scores and potentially obstructing rather than facilitating patient care.

Without a decision support system acting as a coding motivator and facilitator during the consultation, the majority of EHR clinical notes contained either no coded data at all or a single code. This represents a significant missed opportunity for the generation of new knowledge from routine data. Furthermore, preferentially coding symptoms that the physician considers relevant to the diagnosis (symptoms already known to predict the disease) can artificially inflate symptom positive predictive value, undermining epidemiological research.^9^ Thus, there is a distinct possibility that the EHR serves as a consistent positive amplification of known facts about symptom predictors for diagnoses but bears less relationship to the reality of patient experience. It is therefore a poor foundation for new clinical prediction rules.

Some may argue that documentation which is focused on the diagnosis makes for a better, more concise clinical record; while documentation that also contains information unrelated to the final diagnosis (eg, symptoms and signs that the clinician elicited while exploring other diagnoses), is suboptimal, as it can add noise and lead to false positive associations between symptoms and diagnoses. This argument is predicated on the assumption that the diagnosis is correct. However, diagnoses are often wrong. One in 20 US outpatients are estimated to be affected by diagnostic error, which translates to about 12 million adults every year.^22^ Primary care is considered a high-risk area for diagnostic error due to high patient volume, early and thus undifferentiated disease presentation, diagnosis unfolding over several episodes of care, lack of access to diagnostic tests, and low signal-to-noise ratio where nonsevere or self-limiting illness is common.^23–25^ In addition, less selective documentation (ie, which is not solely focused on the diagnosis but reflects what was observed, said and done during the consultation) can be useful to other providers caring for the patient who may seek to understand the patient’s clinical background and help reappraise the situation when treatments do not work.

Preferentially recording information related to the final diagnosis may result from both predecisional^26^ and postdecisional^13^ cognitive processes that satisfy our need for coherent judgments. Clinicians start to formulate diagnostic impressions within seconds in a consultation.^27^ These early diagnostic impressions drive highly selective information gathering and interpretation and can determine the eventual diagnosis and management.^18^^,^^28^ After a diagnosis has been made, and since most physicians document only at the end of the consultation, the documentation can be subject to memory gaps and information distortions. For example, Arkes and Harkness observed such postdiagnosis distortions: study participants mistakenly recognized symptoms related to the diagnosis, even though they were seeing them for the first time; and were more confident in their recognition of diagnosis-related symptoms than diagnosis-unrelated symptoms, despite having seen both before.^29^ This illustrates well the phenomenon that, after an event or response, we retain in memory a coherent representation that does away with the inconsistencies and uncertainties experienced at the time of the response.^19^^,^^30^ For this reason, as long ago as 1980, Arkes and Harkness advised the diagnostician “to record not only the diagnosis but also all symptoms actually observed” and argued that revising an erroneous diagnosis may be hampered “unless the original symptoms are carefully recorded.”^29^

Previous research has provided some evidence that selective recording of information can happen with coded data.^9^ Here, we provide evidence that free-text data may also suffer from selective recording. Previous studies have linked biases in the documentation to the way the EHR is structured to support billing.^6^ Here we show how physicians’ cognitive biases during diagnosis can translate to statistical biases in the EHR documentation and how documentation can improve with the use of a carefully designed DSS. We found less selective (ie, less diagnosis-focused), documentation for both codes and free text in the DSS-supported consultations. This suggests that the early prompts GPs received in the form of a list of diagnostic suggestions led them to explore more diagnostic possibilities, elicit more information—not necessarily related to their final diagnosis—and document that information in either code or free text, resulting in a more complete record.

The primary purpose of the clinical record is to support clinical care, and a more complete record certainly fulfils this purpose better. Secondary purposes are to support epidemiological research, service management, auditing, and billing. All these uses of data can benefit from EHR systems and integrated tools that are designed with awareness of potential cognitive bias and are able to facilitate a more complete and less selective recording of observations in the clinical encounter.

## FUNDING

The study is funded by a Cancer Research UK project grant (C37891/A25310). The original study (DSS evaluation study) was funded by the European Commission DG INFSO (FP7 247787).

## AUTHOR CONTRIBUTIONS

All authors contributed substantially to the design and conduct of the study. In addition, OK performed the statistical analyses and drafted the manuscript, CT prepared the data for analyses, and BCD contributed to the writing of the manuscript.
